# Small airway remodeling in diabetic and smoking chronic obstructive pulmonary disease patients

**DOI:** 10.18632/aging.103112

**Published:** 2020-05-05

**Authors:** Nan Wu, Zhenchao Wu, Jian Sun, Mengdie Yan, Bingbing Wang, Xintong Du, Yi Liu

**Affiliations:** 1 Department of Pulmonary and Critical Care Medicine, Shandong Provincial Hospital Affiliated to Shandong University, Jinan 250021, Shandong, P.R. China; 2 Department of Pulmonary and Critical Care Medicine, Shandong Provincial Hospital Affiliated to Shandong First Medical University, Jinan 250000, Shandong, P.R. China; 3 Cheeloo College of Medicine, Shandong University, Jinan 250021, Shandong, P.R. China

**Keywords:** chronic obstructive pulmonary disease, diabetes mellitus, epithelial-mesenchymal transition, TGF-β/Smad pathway

## Abstract

Diabetes mellitus can reinforce the small airway dysfunction of chronic obstructive pulmonary disease (COPD) patients. The epithelial-mesenchymal transition (EMT) that is associated with small airway remodeling is activated in the airway epithelial cells (AECs) of both COPD patients and diabetic patients. Transforming growth factor β (TGF-β) can induce EMT via the TGF-β/Smad pathway. We found that the small airway dysfunction and airflow limitations were worse in COPD patients with a history of smoking or diabetes than in simple COPD patients, and were even worse in COPD patients with both histories. Pulmonary ventilation tests in rats confirmed these findings. EMT and the TGF-β/Smad pathway were activated in the AECs of rats with COPD or diabetes, and the combination of COPD and diabetes amplified those effects, as indicated by downregulation of Zo1 and upregulation of vimentin, TGF-β and Smad4 in immunohistochemical experiments. Twenty-four-hour treatment with 25 mM glucose and/or 1% cigarette smoke extract upregulated vimentin, TGF-β, Smad2/3/4 and p-Smad2/3, but downregulated Zo1 in AECs. Suppressing the TGF-β/Smad pathway prevented EMT activation and small airway remodeling following cigarette smoke exposure and hyperglycemia. Thus, cigarette smoke and high glucose exposure induces EMT via the TGF-β/Smad pathway in AECs.

## INTRODUCTION

Chronic obstructive pulmonary disease (COPD) is the fourth leading cause of death worldwide, and is predicted to rank third in 2020, laying a heavy burden on national financial systems [1]. One major pathophysiology of COPD is inflammation and narrowing of the peripheral airways, which reduces the forced expiratory volume in 1 second (FEV1) [2]. Hence, the main COPD treatments are inhaled glucocorticoids to suppress inflammation and bronchodilators to improve spirometric indicators such as the FEV1. However, even when receiving optimal medical therapy, many patients with COPD continue to experience distressing breathlessness and an impaired exercise capacity, and there is no evidence that any of the existing medications can prevent long-term declines in pulmonary ventilation. Therefore, new molecules or signaling pathways contributing to COPD must be identified so that new therapeutic regimens can be developed.

Numerous diseases have been found to coexist with COPD, including cardiovascular disease, osteoporosis, lung cancer and so on [3–5]. A clinical trial demonstrated that diabetes is also an important complication of COPD [6]. Approximately 10% of diabetic patients have COPD, and the prevalence of diabetes in COPD patients is 8% higher than that in the general population [7]. In a recent clinical study, COPD patients with concurrent diabetes exhibited worse pulmonary ventilation test (FEV1) results and prognoses than those with COPD alone. This suggests that there is a common mechanism for these two diseases, leading to a worse prognosis when both are present. However, it remains unclear how these two diseases interact.

COPD is a complex condition, and patients frequently exhibit both airway and lung parenchymal damage. The earliest changes take place in the small airways, where most of the airflow limitation occurs. Cigarette smoke (CS), the major pathogenic factor of COPD, has been confirmed to activate the epithelial-mesenchymal transition (EMT), defined as the loss of epithelial characteristics and the gain of mesenchymal characteristics [8]. The EMT is a remarkable process in which transitioning motile epithelial cells digest the basement membrane to become sub-epithelial fibroblasts, which can contribute to fibrosis by releasing extracellular matrix proteins. These fibroblasts are also thought to stimulate the epithelium to further induce EMT, creating a vicious cycle of small airway narrowing and airflow obstruction known as airway remodeling. Multiple signaling pathways have been found to trigger EMT, including those involving tyrosine kinase receptors (epidermal growth factor, fibroblast growth factor, platelet-derived growth factor, insulin-like growth factor, etc.), integrins, Wnt, nuclear factor (NF)-κB and transforming growth factor β (TGF-β) [9].

Chen et al. demonstrated that airway TGF-β expression was greater in streptozotocin-induced diabetic gerbils than in healthy gerbils, and was associated with EMT activation in airway epithelial cells (AECs) [10]. These findings indicated that EMT also contributes to diabetes-induced lung injury. Thus, in this study, we measured the small airway function of patients with COPD, diabetes or both, and evaluated its association with EMT in AECs. We also performed cell and animal experiments to determine the involvement of the TGF-β/Smad pathway in EMT during COPD and diabetes. Our findings may partly explain the higher prevalence of COPD in diabetic patients.

## RESULTS

### Baseline data of COPD patients

All the clinical data from 347 COPD patients, including their baseline characteristics (gender, age, smoking status, body mass index, fasting blood glucose, etc.) and pulmonary ventilation test results (residual volume [RV]/total lung capacity [TLC], FEV1/forced vital capacity [FEV1/FVC], FEV1%pred [the patient’s FEV1% divided by the average FEV1% in the population], peak expiratory flow [PEF], the forced expiratory flow when 25%, 50% or 75% of the FVC had been exhaled [FEF25, FEF50 and FEF75], maximal mid-expiratory flow [MMEF] and so on), were collected from hospital record databases. We divided the COPD patients into four subgroups – simple (non-smoking/non-diabetic COPD patients), smoking, diabetic, and smoking diabetic – and calculated the mean or median of each indicator in the different groups (Table 1).

**Table 1:** Baseline characteristics from 347 COPD patients (Total Sample; Simple, Smoking, Diabetes, Smoking with Diabetes Group).

**COPD patients**	**Total sample**	**Simple group**	**Smoking group**	**Diabetes group**	**Smoking with diabetes group**	**p value**
cases, n	347	98(28.24%)	153(44.09%)	36(10.37%)	60(17.29%)	
Baseline Characteristics						
gender(M/F)	261/86	45/53	142/11	18/18	56/4	<0.01
age, years	67.07±9.26	66.87±9.98	66.42±9.41	69.58±8.44	67.55±7.94	0.34
length of stay, days	11.16±6.59	12.14±6.50	10.74±7.32	9.97±5.62	11.33±5.05	0.05
non-smokers	134(38.62%)	98(100.00%)	-	36(100.00%)	-	
ex-smokers	104(29.97%)	-	68(44.44%)	-	36(60.0%)	<0.01
current smokers	109(31.41%)	-	85(55.56%)	-	24(40.0%)	
smoking-index	908.17±564.96	-	896.47±550.73	-	938.00±603.51	0.83
BMI	25.45±3.78	24.57±4.83	25.36±2.81	25.95±4.19	25.19±3.80	0.87
fasting blood glucose, mmol/L	6.14±1.56	5.41±0.65	5.47±0.67	8.10±1.82	7.88±1.72	<0.01
Pulmonary Ventilation Function						
TLC, %	76.09±16.31	77.21±18.24	76.84±15.69	72.47±13.65	74.24±15.77	0.36
RV/TLC, %	138.94±31.10	132.41±24.92	139.90±34.97	142.51±30.25	145.71±28.88	0.03
FEV1, L	1.19±0.49	1.25±0.53	1.24±0.49	1.04±0.51	1.08±0.35	0.01
FEV1%pred, %	46.55±16.37	54.08±15.88	44.86±16.37	46.13±17.03	38.84±11.47	<0.01
FEV1/FVC, %	54.05±10.27	59.46±7.74	51.87±10.43	54.99±10.62	50.15±9.78	<0.01
PEF, %	42.14±16.97	45.92±17.40	42.12±17.75	35.80±15.26	39.80±13.68	0.01
Small Airway Function						
FEF25, %	21.71±12.55	26.61±12.95	20.71±12.62	21.11±13.84	16.61±7.46	<0.01
FEF50, %	17.58±9.98	21.10±10.14	17.00±9.88	17.77±11.85	13.22±6.25	<0.01
FEF75, %	25.21±15.44	30.28±17.80	22.76±11.39	28.48±21.08	21.62±14.69	<0.01
MMEF, %	18.05±9.43	21.22±10.10	17.36±9.27	17.86±10.39	14.64±6.10	<0.01
FEF75/85, %	48.15 (30.90-78.83)	52.00 (35.30-83.10)	47.10 (29.13-77.80)	47.30 (34.45-217.30)	39.75 (29.28-61.45)	0.23
MMEF/FVC, %	26.63±8.94	27.48±8.52	22.13±8.78	24.76±8.88	20.49±7.91	<0.01

Note: Data presented as median (IQR) unless specified.

Abbreviations: BMI, Body Mass Index; TLC, Total Lung Capacity; RV, Residual Volume; FEV1, Forced Expiratory Volume in 1 second; FVC, Forced Vital Capacity; PEF, Peak Expiratory Flow; FEF, Forced Expiratory Flow; MMEF, Maximum Mid-Expiratory Flow.

### The degree of airway obstruction and airflow limitation differed among the COPD subgroups

**Figure 1:**
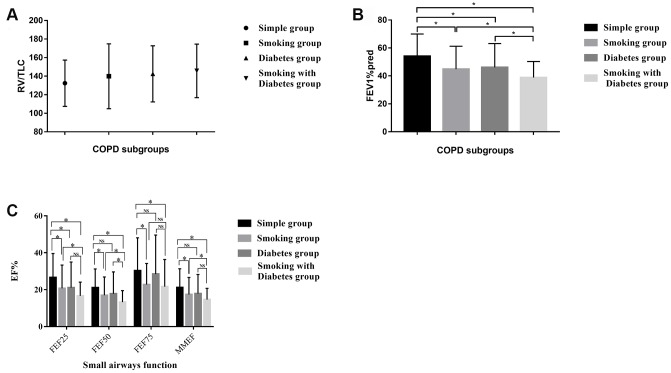
(**A**) The distribution of the RV/TLC% in the different COPD subgroups. (**B**) The distribution of the FEV1%pred in the different COPD subgroups. (**C**) Small airway disorders identified using the pulmonary ventilation test in the different COPD patients. * indicates a significant difference between two groups; NS indicates no significant difference between two groups.

The most important and widely accepted indicators used to detect persistent airflow limitations in COPD patients are FEV1%pred, FEV/FVC (both measured after bronchodilator inhalation) and RV/TLC. RV/TLC reflects the proportion of the residual volume of the lungs, which is relevant to airway obstruction. RV/TLV tended to be lower in the simple COPD group than in the other groups, though the differences were not significant (Table 2). These results suggested that the co-existence of hyperglycemia-induced lesions worsened the RV of COPD patients (Figure 1A). FEV1 was used to assess the severity of airflow limitations in accordance with the Global Initiative for Chronic Obstructive Lung Disease (GOLD) system. The airflow limitations were more serious in smoking COPD patients and diabetic COPD patients than in simple COPD patients, and were the worst in smoking diabetic COPD patients (Figure 1B).

**Table 2:** Small airways function was compared with each two groups by RV/TLC, FEV1%pred, FEF25, FEF50, FEF75, MMEF in COPD patients.

**COPD subgroups**	**exposure & outcomes**	**simple group vs. smoking group**	**simple group vs. diabetes group**	**simple group vs smoking with diabetes group**	**smoking group vs. smoking with diabetes group**	**diabetes group vs. smoking with diabetes group**
p value	RV/TLC	0.06	0.07	<0.01	0.29	0.64
	FEV1%pred	<0.01	0.01	<0.01	<0.01	0.03
	FEF25, %	<0.01	0.03	<0.01	<0.01	0.08
	FEF50, %	<0.01	0.11	<0.01	<0.01	0.04
	FEF75, %	<0.01	0.64	<0.01	0.56	0.08
	MMEF,%	<0.01	0.09	<0.01	0.02	0.10

### Pulmonary ventilation tests revealed small airway disorders in smoking diabetic COPD patients

We used the FEF25, FEF50, FEF75 and MMEF values from the pulmonary ventilation test to assess the presence of small airway disorders. The respiratory community has deemed that a patient has a small airway disorder if any two of the above indicators are less than 60%. All 347 of our included patients had small airway disorders, but some of them exhibited a more serious condition. The smoking COPD patients and diabetic COPD patients displayed a significantly greater degree of damage in their small airways than the simple COPD patients. The worst small airway function was observed in diabetic COPD patients who were former or current smokers (Table 2 and Figure 1C).

### Pulmonary ventilation test results were worse in diabetic than in non-diabetic rats

We then assessed the lung ventilation function of control rats, COPD rats (induced by CS), diabetic rats and COPD diabetic rats (Table 3). The tidal volume [box] (TVb) and minute volume [box] (MVb) did not differ among the groups (Table 3). However, the peak expiratory flow [box] (PEFb) and the expiratory flow when 50% of the TV had been expired (EF50) were reduced and the expiratory time (Te) was prolonged in COPD rats, diabetic rats and COPD diabetic rats compared with control rats. COPD diabetic rats had worse pulmonary ventilation test results than COPD or diabetic rats alone. These results implied that diabetes can impair lung ventilation. However, the above indicators were improved in COPD diabetic rats injected with SB431542 (an inhibitor of the activin receptor-like kinase [ALK] receptors ALK5, ALK4 and ALK7).

**Table 3:** The indicators of pulmonary function test and the level of fasting blood glucose in different rat models.

**rats model**	**control**	**smoking**	**diabetes**	**smoking with diabetes**	**smoking with diabetes with inhibitor**	**p value**
case, n	5	5	5	3	3	-
weigh, g	473.1	541	395	519.3	481	-
blood glucose, mmol/L	4.7	4.7	22.2	23.7	19.6	-
WBP Pulmonary Function						
TVb	2.46±0.52	2.92±0.32	2.70±0.27	3.30±0.52	2.82±0.34	0.16
MVb	353.62±13.42	343.53±31.37	279.18±70.20	317.71±74.96	367.92±0.27	0.34
PEFb	15.24±0.67	13.94±0.10	13.47±2.39	12.70±0.00	17.92±0.85	0.02
Te	0.31±0.02	0.35±0.01	0.38±0.06	0.36±0.01	0.28±0.03	0.03
EF50	1.10±0.11	0.97±0.15	0.78±0.21	0.77±0.00	1.21±0.05	0.01

Note: TVb: Tidal Volume (box) volume inhales; MVb: Minute Volume (box) the rate of ventilation; PEFb: Peak Expiratory Flow (box); Te: Expiratory Time; EF50: Expiratory flow at the point 50% of TV is expired

**Figure 2:**
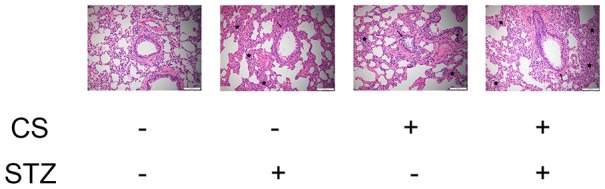
**CS-induced COPD and streptozotocin-induced diabetes damaged the lungs of rats.** Hematoxylin and eosin staining of bronchioles from control, COPD, diabetic and COPD diabetic rats. →: Bronchiole with smooth muscle hyperplasia, globe cell metaplasia and inflammatory cell infiltration; **★**: Hyperplastic parenchyma with inflammatory cell infiltration.

Hematoxylin and eosin staining revealed that the airway lumen diameters were lower in diabetic rats and COPD rats than in control rats, and were the lowest in rats with both diabetes and COPD (Figure 2). These results indicated that diabetes can impair pulmonary ventilation in both healthy and COPD rats, and may be associated with airway remodeling.

### High glucose levels and CS exposure activated EMT in AECs

**Figure 3:**
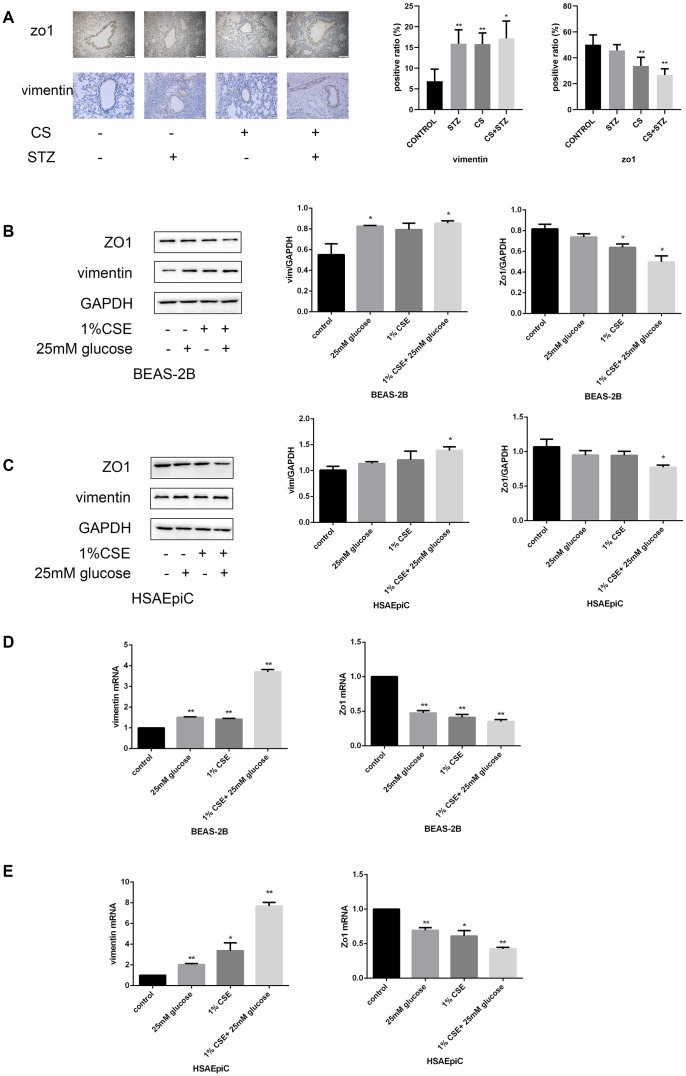
**High glucose levels and CS exposure activated EMT in AECs.** (**A**) Immunohistochemical results and positive ratios of Zo1 and vimentin in control, COPD, diabetic and COPD diabetic rats. (**B**–**E**) AECs were treated with 25 mM glucose, 1% CSE or both for 24 hours. Western blots of Zo1 and vimentin in BEAS-2B (**B**) and HSAEpiC (**C**) cells. qRT-PCR analyses of *Zo1* and *vimentin* in BEAS-2B (**D**) and HSAEpiC (**E**) cells. * p<0.05, ** p<0.01 compared with the control group.

EMT in AECs is a key contributor to airway remodeling. To investigate the mechanism(s) whereby diabetes and COPD induced airway remodeling, we measured markers of the EMT both *in vivo* and *in vitro*. Immunohistochemical staining of the airways indicated that Zo1 (an epithelial biomarker) was downregulated and vimentin (a mesenchymal biomarker) was upregulated in rats with diabetes or COPD, and the combination of both diseases amplified these effects (Figure 3A).

We then performed an *in vitro* experiment by treating AECs (BEAS-2B and HSAEpiC cells) with 25 mM glucose and/or 1% CS extract (CSE). These treatments reduced Zo1 and increased vimentin protein levels in both BEAS-2B and HSAEpiC cells (Figure 3B, 3C), indicating that high glucose levels and CS exposure induced EMT. These conclusions were supported by quantitative real-time PCR (qRT-PCR) analyses of the corresponding mRNA levels (Figure 3D, 3E).

### High glucose levels and CS exposure activated the TGF-β signaling pathway

To determine the molecular pathway through which diabetes and COPD activated EMT in AECs, we measured the expression of TGF-β signaling molecules in rat airways and AECs. As shown in Figure 4A–4C, high glucose levels and/or CS exposure clearly induced TGF-β expression both *in vivo* and *in vitro*. Smad4 was also activated in the airways of the COPD, diabetic and combination rat models (Figure 4D). In AECs treated with 25 mM glucose and/or 1% CSE, Smad2/3/4 mRNA and protein levels and phosphorylated (p)-Smad2/3 levels were greater than those in control AECs (Figure 4E–4H), further indicating that the TGF-β signaling pathway was activated *in vitro*.

**Figure 4A–E:**
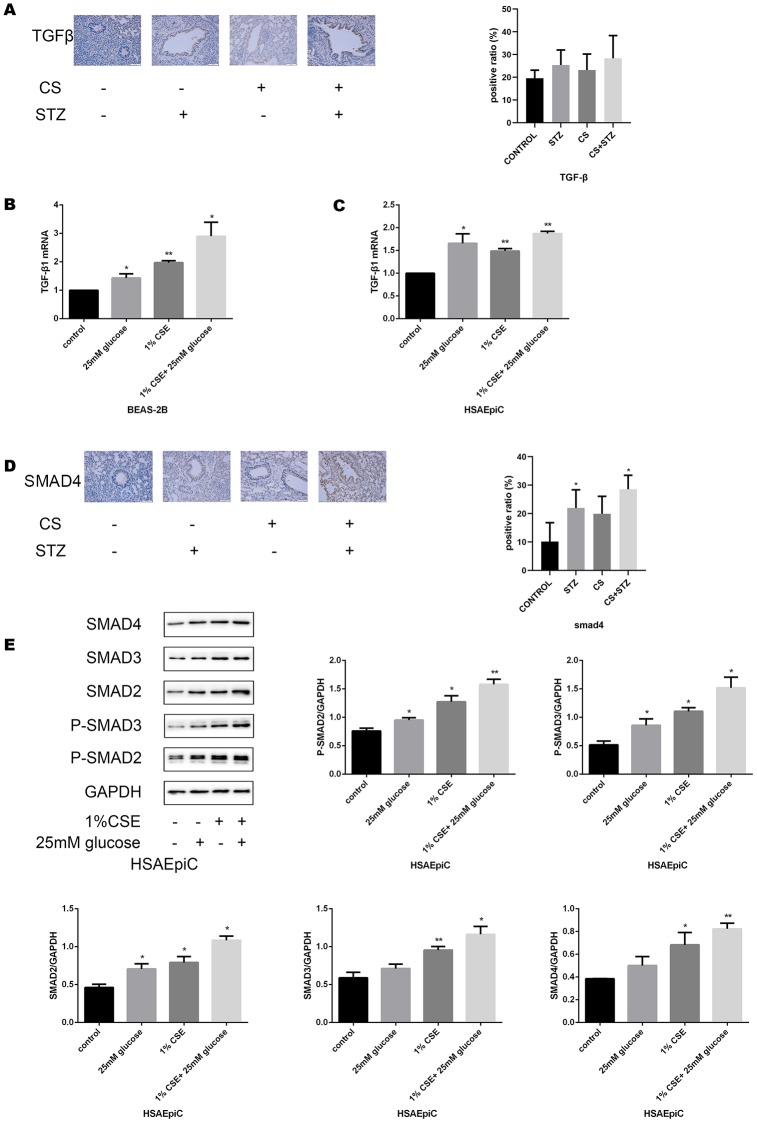
**High glucose levels and CS exposure activated the TGF-β signaling pathway.** (**A**) Immunohistochemical results and positive ratios of TGF-β1 in control, COPD, diabetic and COPD diabetic rats. (**B**, **C**) AECs were treated with 25 mM glucose, 1% CSE or both for 24 hours. qRT-PCR results of TGF-β1 in BEAS-2B (**B**) and HSAEpiC (**C**) cells. (**D**) Immunohistochemical results and positive ratios of Smad4 in CS-induced COPD, diabetic and COPD diabetic rats. (**E**–**H**) AECs were treated with 25 mM glucose, 1% CSE or both for 24 hours.

**Figure 4F–H:**
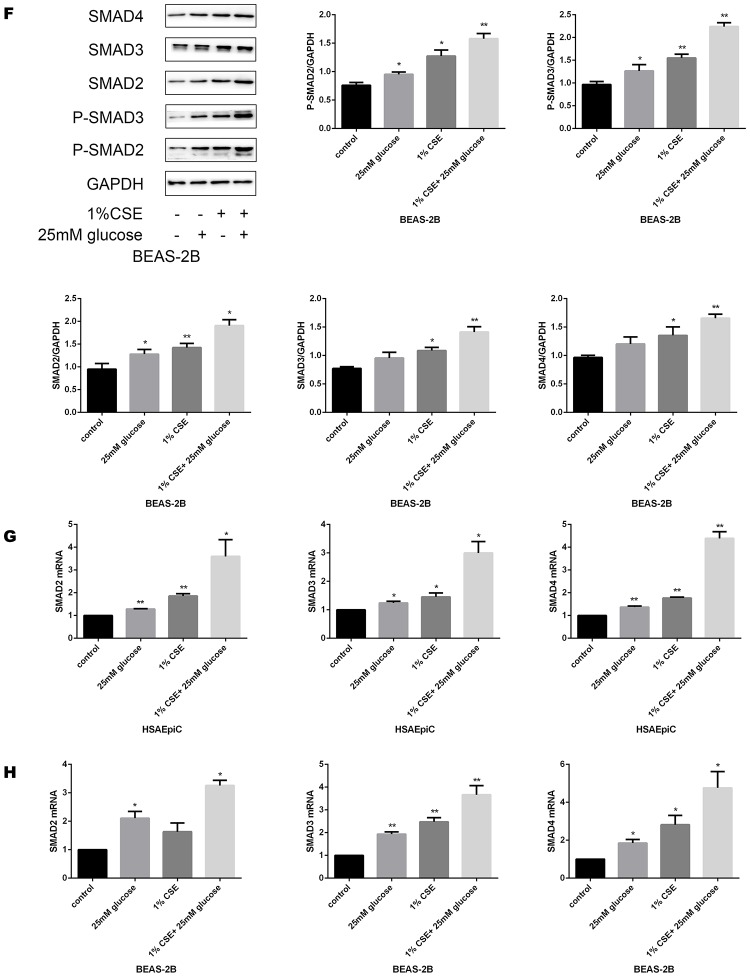
**High glucose levels and CS exposure activated the TGF-β signaling pathway.** Western blot results of Smad2, Smad3, Smad4, p-Smad2 and p-Smad3 in BEAS-2B (**F**) and HSAEpiC (**E**) cells. qRT-PCR results of Smad2, Smad3 and Smad4 in BEAS-2B (**H**) and HSAEpiC (**G**) cells. * p<0.05, ** p<0.01 compared with the control group.

### TGF-β signaling pathway activation/inhibition determined the EMT level in AECs

The TGF-β signaling pathway has different functions in different cell types and conditions. To determine the function of this pathway in AECs, we treated cells with a TGF-β signaling pathway activator (TGF-β, 230 pg/mL, 2 hours) or inhibitor (SB431542, 10 μg/mL, 30 minutes). Western blotting and qRT-PCR analyses indicated that activating the TGF-β signaling pathway with TGF-β induced EMT in AECs with or without 1% CSE, as evidenced by reduced Zo1 expression and increased vimentin expression (Figure 5A–5D). Inhibiting the TGF-β pathway with SB431542 had the opposite effects (Figure 5E–5H).

**Figure 5A–D:**
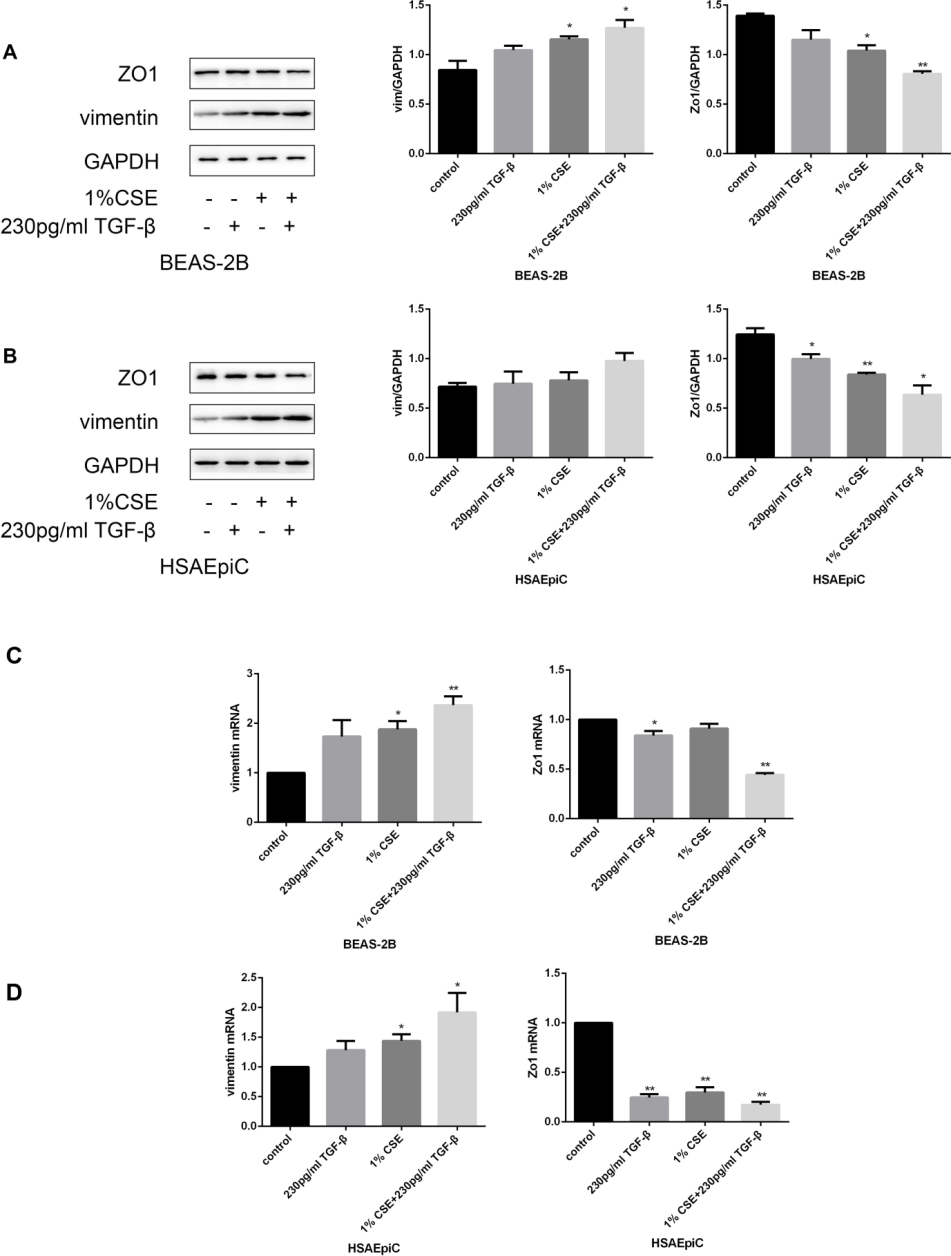
**The TGF-β signaling pathway determined the EMT level.** (**A**–**D**) AECs were treated with 230 pg/mL TGF-β1 for 2 hours. Western blots of Zo1 and vimentin in BEAS-2B (**A**) and HSAEpiC (**B**) cells. qRT-PCR analyses of Zo1 and vimentin in BEAS-2B (**C**) and HSAEpiC (**D**) cells.

**Figure 5E–H:**
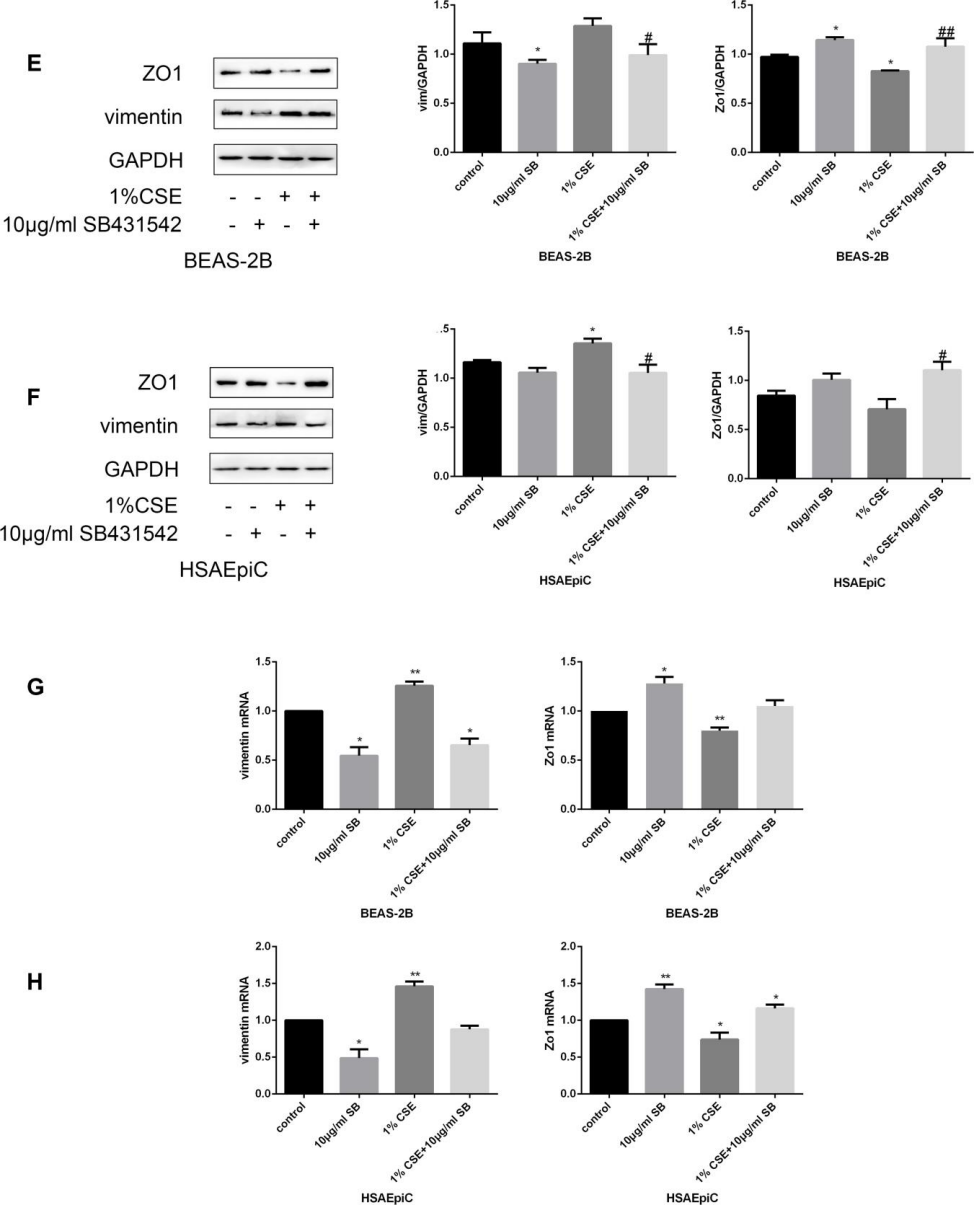
**The TGF-β signaling pathway determined the EMT level.** (**E**–**H**) AECs were treated with 10 μg/mL SB431542 for 30 minutes. Western blots of Zo1 and vimentin in BEAS-2B (**E**) and HSAEpiC (**F**) cells. qRT-PCR analyses of Zo1 and vimentin in BEAS-2B **(G**) and HSAEpiC (**H**) cells. * p<0.05, ** p<0.01 compared with the control group; # p<0.05, ## p<0.01 compared with the CSE group SB: SB431542.

### High glucose levels and CS exposure induced EMT through the TGF-β signaling pathway

The above results separately demonstrated that high glucose levels and CS exposure could activate EMT and the TGF-β signaling pathway in AECs, and that the TGF-β signaling pathway could activate EMT in AECs. Thus, we further investigated whether the TGF-β signaling pathway induced EMT in AECs upon high glucose and CS exposure. When COPD diabetic rats were injected with the TGF-β inhibitor SB431542, their epithelia were less injured than those of rats injected with the buffer (Figure 6A). Immunohistochemical analyses of the airways indicated that EMT was suppressed in SB431542-injected rats compared with buffer-injected rats, as evidenced by lower vimentin expression and higher Zo1 expression (Figure 6B). *In vitro* experiments in AECs treated with 25 mM glucose and 1% CSE supported these conclusions, as Zo1 expression increased and vimentin expression decreased after SB431542 treatment (Figure 6C–6F), while TGF-β treatment had the opposite effects (Figure 6G–6J).

**Figure 6A–E:**
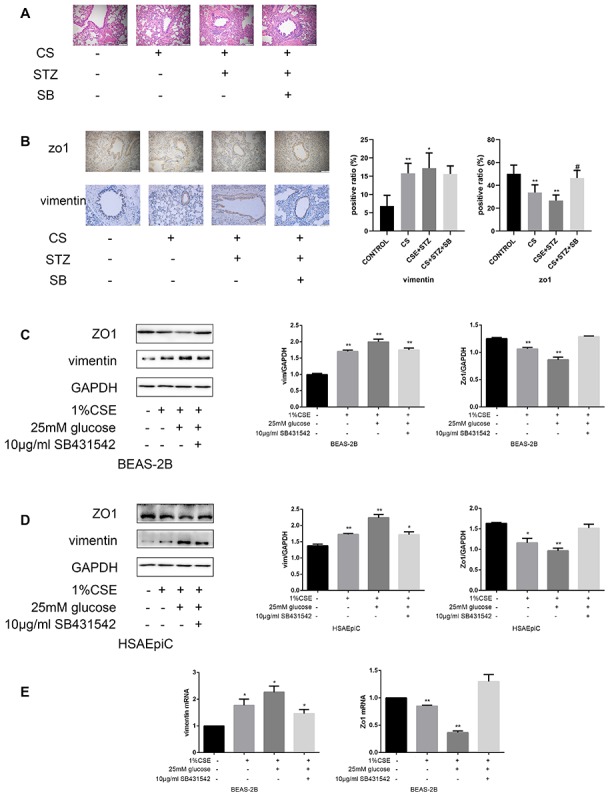
**High glucose levels and CS exposure induced EMT through the TGF-β signaling pathway.** (**A**) Hematoxylin and eosin staining of bronchioles from control, COPD, COPD diabetic and SB431542-injected COPD diabetic rats. (**B**) Immunohistochemical results and positive ratios of Zo1 and vimentin in control, COPD, COPD diabetic and SB431542-injected COPD diabetic rats. (**C**–**F**) AECs were treated with 25 mM glucose and 1% CSE for 24 hours, and SB431542 was added 30 minutes before further treatment. Western blots of Zo1 and vimentin in BEAS-2B (**C**) and HSAEpiC (**D**) cells. qRT-PCR analyses of Zo1 and vimentin in BEAS-2B (**E**) cells.

**Figure 6F–J:**
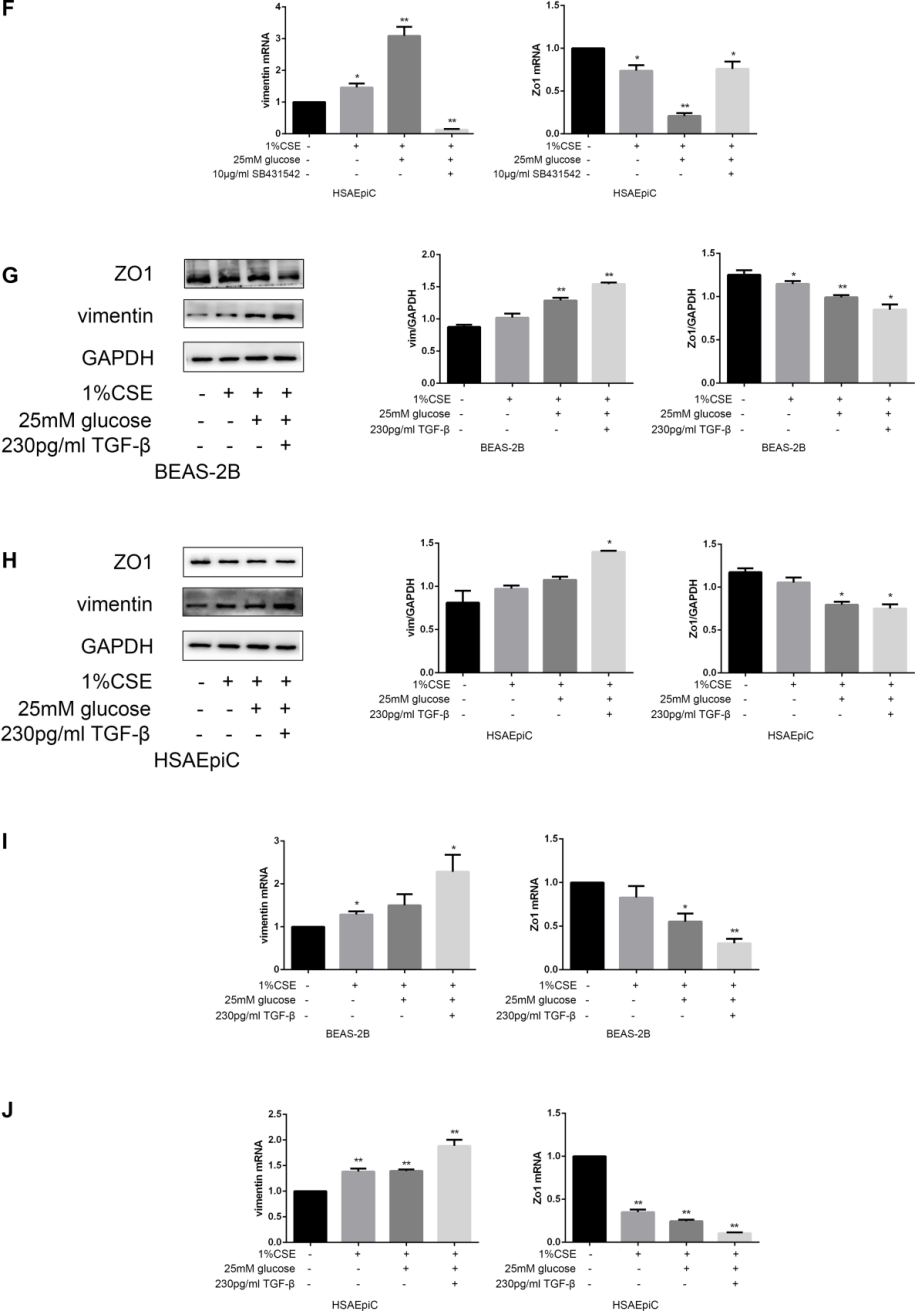
**High glucose levels and CS exposure induced EMT through the TGF-β signaling pathway.** qRT-PCR analyses of Zo1 and vimentin in HSAEpiC (**F**) cells. (**G**–**J**) AECs were treated with 25 mM glucose and 1% CSE for 24 hours, and TGF-β was added 2 hours before the cells were harvested. Western blots of Zo1 and vimentin from BEAS-2B (**G**) and HSAEpiC (**H**) cells. qRT-PCR analyses of Zo1 and vimentin in BEAS-2B (**I**) and HSAEpiC (**J**) cells. * p<0.05, ** p<0.01 compared with the control group; # p<0.05, ## p<0.01 compared with the CSE group.

## DISCUSSION

Previous clinical results have demonstrated that type 2 diabetes increases the severity of COPD, that type 2 diabetes is more prevalent in COPD patients than in the general population (18.7% vs. 10.5%) and that approximately 10% of type 2 diabetic patients also have COPD [11, 12]. However, the mechanisms responsible for the comorbidity of COPD and type 2 diabetes have not been described. Hyperglycemia, one of the most important characteristics of type 2 diabetes, may be one such mechanism.

As the first line of protection for the airways and lungs, the epithelium is an essential barrier against outer irritants [13, 14]. However, when AECs are overexposed to irritants such as CS and other environmental factors, they can undergo EMT, characterized by the loss of polarity, reduced epithelial marker expression, loss of tight junctions, and increased mesenchymal characteristics such as motility, depolarized cytoskeletal arrangements, vimentin and alpha smooth muscle actin overexpression and so on [15–17]. Various studies by Sohal, Soltani, Mahmood and others have demonstrated that small airway dysfunction is associated with EMT [18–24]. Mahmood et al. reported that increased levels of the mesenchymal markers S100A4 and vimentin were associated with reduced lung function, indicating that the EMT may be a key contributor to COPD pathology [19, 25]. Milara et al. demonstrated that EMT was induced in COPD patients, probably due to CS [8]. We previously found that siRNA against human antigen R prevented the CS-induced downregulation of E-cadherin and upregulation of vimentin and zinc finger E-box binding homeobox 1 (ZEB1) in AECs *in vitro* [26], suggesting that human antigen R partially enhances the EMT and post-transcriptionally regulates the airway epithelium in COPD. Other aspects of COPD pathogenesis include large airway squamous metaplasia, mucus hypersecretion, smooth muscle hyperplasia, small airway fibrosis and airway wall thickening. These physiological processes could narrow the airway lumen [27].

Chen et al. reported that EMT was activated in a streptozotocin-induced diabetic gerbil model [10]. Hence, we hypothesized that hyperglycemia could reinforce the effects of CS exposure by activating EMT in AECs, further impairing pulmonary ventilation in COPD patients. Our study revealed that diabetic smoking COPD patients had worse small airway function than smoking COPD patients. Rats with diabetes, COPD or both exhibited worse airway epithelial function, greater vimentin expression and lower Zo1 expression than control rats, indicating that the EMT was activated in type 2 diabetes and COPD. Our *in vitro* results in both BEAS-2B and HSAEpiC cells indicated that 25 mM glucose or 1% CSE exposure for 24 hours could upregulate EMT by increasing vimentin expression and reducing Zo1 expression. Moreover, high glucose treatment and CSE exposure had synergistic effects, supporting our hypothesis.

Milara et al. reported that CSE induced EMT in primary bronchial epithelial cells by stimulating reactive oxygen species production, which enhanced TGF-β1 expression and reduced cyclic adenosine monophosphate levels [28, 29]. Our experiments revealed that high glucose levels reinforced the effects of CS exposure by activating the TGF-β signaling pathway, while inhibiting the TGF-β signaling pathway suppressed the activation of EMT upon high glucose and CS exposure. These results suggested that both hyperglycemia and COPD stimulate EMT through the TGF-β signaling pathway.

In the TGF-β signaling pathway, after the activation of the membrane receptor serine/threonine kinase complex, the transcription factors Smad2 and Smad3 are phosphorylated and transferred to the nucleus, where they bind to Smad4 to regulate the expression of target genes [30, 31]. Chen et al. found that diabetic gerbils had a smaller alveolar airspace and thicker septa than controls, and that their lung cells displayed higher TGF-β levels and a transformation from epithelial to mesenchymal characteristics [10]. However, their results only indicated that high glucose levels upregulated TGF-β. We further demonstrated that high glucose levels activated the TGF-β/Smad pathway, as indicated by the increased Smad2/3/4 expression and Smad2/3 phosphorylation in glucose-exposed AECs and the increased Smad4 expression in the airways of diabetic rats.

Milara et al. reported that primary AECs isolated from smokers or COPD patients expressed higher levels of mesenchymal markers (alpha smooth muscle actin, vimentin and type 1 collagen) and lower levels of epithelial markers (E-cadherin, Zo1 and cytokeratin 5/18) than those from non-smokers [8]. Their *in vitro* experiments also demonstrated that EMT was activated in AECs exposed to CSE for 72 hours, as evidenced by the release of TGF-β, the phosphorylation of Smad3 and extracellular signal-regulated kinase ½, and the downregulation of cyclic adenosine monophosphate [8]. Our results revealed that EMT was activated in AECs exposed to 1% CSE for 24 hours, as indicated by the upregulation of Smad2/3/4 and p-Smad2/3 in both BEAS-2B and HSAEpiC cells. Furthermore, Smad4 was overexpressed in the airways of rats with COPD. Glucose reinforced the effects of CS on EMT.

The function of the TGF-β signaling pathway varies according to the cell type, culture conditions and surrounding environment [32]. Hence, we explored the function of this pathway under our experimental conditions by using the TGF-β pathway inhibitor SB431542 and the activator TGF-β. Inhibiting the TGF-β pathway suppressed EMT in AECs, while activating the TGF-β pathway had the opposite effect. Moreover, SB431542 suppressed the induction of EMT by the combination of glucose and CS both *in vitro* and *in vivo*, while TGF-β treatment enhanced EMT under these conditions. These results confirmed that high glucose and CS exposure can activate EMT in AECs through the TGF-β signaling pathway.

## MATERIALS AND METHODS

### Study population

In this retrospective study, data from 347 patients with new discharge diagnoses of COPD based on the GOLD 2014 criteria were obtained from the hospital record database of Shandong Provincial Hospital from January 1^st^, 2014 to December 31^st^, 2017. Patients were only included if they were over 40 years old, had records of pulmonary ventilation test results and were alive when discharged from the hospital.

The 347 patients were divided into four subgroups: a) the simple COPD group: COPD patients who were non-smokers without diabetes, b) the smoking COPD group: COPD patients who were current smokers or ex-smokers but did not have diabetes, c) the diabetic COPD group: COPD patients who were non-smokers but had diabetes, and d) the smoking diabetic COPD group: COPD patients who were current smokers or ex-smokers and had diabetes.

As this was a retrospective study, there was no randomization, no exploration of new treatments and no potential harm to the patients.

### Animals

Adult male Wistar rats weighing around 200 g were used for all the experiments. The rats were raised in a room with a 12-hour/12-hour dark/light cycle, controlled temperature (22 ± 2 °C) and humidity (55 ± 5 %), and free access to food and water. The rats were randomly divided into four groups: control (n=5), COPD (n=5), diabetes (n=5) and COPD combined with diabetes (n=6). The combination group was further divided into the SB431542-injected group (n=3) and the buffer-injected group (n=3). The models and treatments are described in greater detail below.

### Type 2 diabetes model

Streptozotocin, an antineoplastic agent that is particularly toxic to the mammalian insulin-producing pancreatic beta cells, is widely used to induce diabetes in animals [33, 34]. In our experiments, rats were fed a high-fat, high-glucose diet for one month before being intraperitoneally injected with streptozotocin (35 mg/kg), and kept feeding such diet till they were sacrificed [35]. One week after the injection, blood glucose was measured through the tail vein, and diabetes was established if the blood glucose level was greater than 11.1 mmol/L and the rat exhibited typical diabetes symptoms. Blood was drawn every two weeks, and streptozotocin was reinjected (35 mg/kg) if the blood glucose level dropped below 11.1 mmol/L. The non-diabetic group was intraperitoneally injected with buffer only.

### COPD model

We established the COPD rat model by exposing rats to CS for 30 minutes twice a day, with an interval of at least 6 hours between exposures. The exposure procedure was performed five times per week for 12 weeks. The non-COPD groups were placed in a cage with free access to fresh air. After the exposure period, all the rats were fed in the room previously described, with free access to fresh air.

We used a pulmonary ventilation test instrument for small animals (Fine Pointe Buxco WBP 601-1400-001, Data Sciences International, MN, USA) to measure the TVb, MVb, PEFb, Te and EF50 values of the rats. TVb is the tidal volume (box); MVb is the minute volume (box), the rate of ventilation; PEFb is the peak expiratory flow (box), the estimated maximum expiratory flow that occurs in one breath; Te is the expiratory time, the time spent exhaling during each breath, from the start to the end of expiration; and EF50 is the expiratory flow at the point when 50% of the tidal volume has been expired. The rats were sacrificed after the pulmonary ventilation test was performed, and their lung tissues were obtained for further experiments.

### SB431542 injection

Ten days before the rats were sacrificed, the SB431542 group was intraperitoneally injected with 2.5 mg/kg SB431542 dissolved in 5% dimethyl sulfoxide, and the buffer group was intraperitoneally injected with 5% dimethyl sulfoxide only [36–38]. The injections were performed every 48 hours for a total of five times.

### Histology and morphometric analyses

The lung tissues were fixed in 4% paraformaldehyde for more than 24 hours, and were embedded in paraffin before being cut into 4.5-μm-thick sections. The sections were stained with hematoxylin and eosin.

### Immunohistochemical staining

The paraffin-embedded lung tissue sections were deparaffinized and dyed overnight. Antigen retrieval was performed with 5 minutes of pressure at a temperature greater than 95 °C in a pressure cooker containing citrate antigen retrieval solution. Then, the sample was cooled down on ice. Nonspecific reactions were blocked with goat serum for 15 minutes at 37 °C. Then, the sections were incubated with primary antibodies against vimentin (Proteintech, 10366-1-AP, Chicago, IL, USA, 1:2000), Zo1 (Proteintech, 21773-1-AP, 1:200), TGF-β (Proteintech, 21898-1-AP, 1:200) or Smad4 (Proteintech, 10231-1-AP, 1:50) for 1 hour at 37 °C. The sections were incubated with the components of a two-step secondary antibody kit (ZSGB-BIO, PV9003, Beijing, China) for 20 minutes each at 37 °C. The samples were viewed under a confocal FV 1000 SPD laser scanning microscope (Olympus, Japan), and positive ratio analyses were performed with ImageJ.

### Cell culture, exposure and treatment

Human bronchial epithelial cells (BEAS-2B) were obtained from Genechem Co. Ltd. (Shanghai, China), and human small airway epithelial cells (HSAEpiC) were obtained from Xinmin Biotechnology Co. Ltd. (Shanghai, China). BEAS-2B cells were cultured in Dulbecco’s modified Eagle’s medium (containing 1 g/L glucose) supplemented with 20% fetal bovine serum (Gibco, 10099-141, Grand Island, NY, USA), 100 U/mL penicillin and 0.1 mg/mL streptomycin (Beyotime, C0222, Shanghai, China). HSAEpiC cells were cultured in RPMI-1640 medium (Gibco, 8118073), supplemented with 10% fetal bovine serum, 100 U/mL penicillin and 0.1 mg/mL streptomycin. The cells were incubated at 37 °C in 5% CO_2_ and 95% air.

The cells were treated with 25 mM glucose for 24 hours, with or without simultaneous exposure to 1% CSE. The CSE was prepared from Daqianmen Brand cigarettes. Each cigarette was bubbled slowly in 10 mL of RPMI-1640 medium until a 100% CSE solution was obtained. The solution was sterile-filtered through a 0.22-μm filter, segregated, sealed and stored at -80 °C. Repeated freezing and thawing was avoided.

To investigate the function of the TGF-β signaling pathway, we treated cells with 230 pg/mL TGF-β1 (Proteintech, HZ-1011) for 2 hours or 10 μg/mL SB431542 (Selleck, S1067, Houston, TX, USA) for 30 minutes before performing further treatments. The cells were starved for 16 hours before being provided complete culture medium for further treatment.

### Western blot

Radioimmunoprecipitation assay buffer supplemented with 1% phenylmethanesulfonyl fluoride was used to lyse the cells. Then, an 8% sodium dodecyl sulfate polyacrylamide gel was used to electrophoretically separate 25 μg of protein from each sample, and the proteins were transferred onto a 0.45-μm polyvinylidene difluoride membrane. The membrane was blocked with 5% fat-free milk (or 5% bovine serum albumin for phosphorylated protein detection) for 1 hour at room temperature, and then was washed with Tris-buffered saline with 0.05% Tween-20 (Solarbio, T8220, China) three times. The membrane was then incubated with primary antibodies against glyceraldehyde-3-phosphate dehydrogenase (Proteintech, 10494-1-AP, 1:10000), vimentin (CST, 5741, Boston, MA, USA, 1:1000), Zo1 (Proteintech, 21773-1-AP, 1:1000), Smad2/3/4 or p-Smad2/3 (CST, 12747, 1:1000) for 16-20 hours at 4 °C. Then, the membrane was washed three times with Tris-buffered saline with Tween-20 before and after being incubated with the secondary antibody (ZSGB-BIO, ZB-2301, 1:5000) for 1 hour at room temperature. Proteins were detected with an ECL Plus detection system (Thermo Scientific, Pittsburgh, PA, USA). Grayscale was detected with ImageJ.

### RNA extraction and qRT-PCR

TRIzol (Invitrogen, USA) was used to extract total RNA from BEAS-2B and HSAEpiC cells, and 2.5 μg of total RNA was reverse-transcribed into cDNA. Then, qRT-PCR was performed with an Ultra SYBR Mixture kit (Cwbio, CW2601M). The sequences of the primers were as follows: Forward-vimentin: 5’-AATGGCTCGTCACCTTCGTG-3’, Reverse-vimentin: 5’-CAGAGAAATCCTGCTCTCCTCG-3’; Forward-Zo1: 5’-GAAATACCTGACGGTGCTGC-3’, Reverse-Zo1: 5’-GAGGATGGCGTTACCCACAG-3’; Forward-TGF-β: 5’-CTGCAAGTGGACATCAACGG-3’, Reverse-TGF-β: 5’-TCCGTGGAGCTGAAGCAATA-3’; Forward-Smad2: 5’-GAGAGCAGAATGGGCAGGAA-3’, Reverse-Smad2: 5’-AGAGCAAGTGCTTGGTATGG-3’; Forward-Smad3: 5’-AGCTGTGTGAGTTCGCCTTC-3’, Reverse-Smad3: 5’-CACAGGAGGTAGAACTGGTGTC-3’; Forward-Smad4: 5’-AAACCATCCAGCATCCACCA-3’, Reverse-Smad4: 5’-TATACTGGCAGGCTGACTTGTG-3’.

**Statistics**

All the data represent the results of three independent experiments. EpiData (Version 3.1) was used to collect and record data. Statistical analyses were performed with SPSS Version 23.0 (SPSS Inc., Chicago, IL, USA) and GraphPad Prism 7 (GraphPad, San Diego, CA, USA). Parametric data are presented as the mean ± the standard error of the mean or range. Non-parametric data are presented as the median (interquartile range). The Kruskal-Wallis test was used for multi-group comparisons, and then the Mann-Whitney U test was used to compare pairs of groups. The chi-squared test (χ2) was used to compare proportions among groups. T tests were used to analyze parametric data. P<0.05 was considered as the threshold of significance for all statistical analyses.

### Study approval

The clinical study was approved by the Medical Ethics Committee of Shandong Provincial Hospital affiliated to Shandong University (Ethical Review of Medical Research on Human Beings No. 2019-001). The animal research protocol was approved by the Medical Experimental Animal Care Commission of Shandong Provincial Hospital affiliated to Shandong University (Ethical Review of Medical Research on Animals No. 2019-019).
